# A Coverage and Slicing Dependencies Analysis for Seeking Software Security Defects

**DOI:** 10.1155/2014/463912

**Published:** 2014-04-02

**Authors:** Hui He, Dongyan Zhang, Min Liu, Weizhe Zhang, Dongmin Gao

**Affiliations:** ^1^School of Computer Science and Technology, Harbin Institute of Technology, Harbin 150001, China; ^2^Department of Computer Science and Technology, University of Science and Technology Beijing, Beijing, China

## Abstract

Software security defects have a serious impact on the software quality and reliability. It is a major hidden danger for the operation of a system that a software system has some security flaws. When the scale of the software increases, its vulnerability has becoming much more difficult to find out. Once these vulnerabilities are exploited, it may lead to great loss. In this situation, the concept of Software Assurance is carried out by some experts. And the automated fault localization technique is a part of the research of Software Assurance. Currently, automated fault localization method includes coverage based fault localization (CBFL) and program slicing. Both of the methods have their own location advantages and defects. In this paper, we have put forward a new method, named Reverse Data Dependence Analysis Model, which integrates the two methods by analyzing the program structure. On this basis, we finally proposed a new automated fault localization method. This method not only is automation lossless but also changes the basic location unit into single sentence, which makes the location effect more accurate. Through several experiments, we proved that our method is more effective. Furthermore, we analyzed the effectiveness among these existing methods and different faults.

## 1. Introduction

Software is the soul of the information systems [1] and plays a crucial role in the information society. High-tech product is often a software-intensive system, from cell phone daily used to Internet applications connected the global. Software has become a part of people's lives. However, software defects seriously affect software quality and software reliability, and it is the major hidden danger of information systems operating stably. Constant software failure and accident make people have to pay much attention to software quality and reliability. So on the one hand, the existence of software defects has brought a great challenge to the safe and reliable operation of information systems. On the other hand, to a greater extent it has affected national security, social stability, and economic development [2].

Software security testing is an extremely challenging task. Statistics show that, in a typical software development project, software testing often accounts for about 50% of the total workload. For some high security and reliability software, testing time even accounts for 60% of the development cycle [3, 4]. As an important part of the automation in the software development process, automated software testing has caused widespread concern. On the other hand, with the continuous development of software technology, new software error or defect types continue to be found. It brings new problems and challenges to software testing technology. Therefore, the research of accurate, high degree of automation software debugging method, on the one hand, can help correctly understand the trigger conditions of the defect and repair defect reasonably. On the other hand, you can greatly reduce the scope of the code to be checking the number of program execution, workload, and cost. And software fault diagnosis is a critical step in the software security testing.

## 2. Software Fault Localization Methods

Software fault localization and diagnosis technology is an important technology in the software defect detection methods. Although the study contains multiple ways, the software only during the test and run-time environment integrated consideration, so software testing in defect detection cannot be replaced. At the same time, the growth of software size and software structure and morphological changes make fault localization and diagnosis in the software testing more difficult. It directly indicates the need for software fault localization and diagnostic techniques.

In the existing research, typical fault localization methods include coverage fault localization and the incremental software debugging (Delta Debugging), based on program slicing fault localization. According to different starting points and analysis of objects, the methods above can be divided into two categories, defined as single execution record fault localization and based on the statistical fault localization [5]. Single execution record fault localization method is execution results oriented. It means that, in the fault localization process, not only do we need to know if the execution result is correct, but also, more importantly, we need to know clearly the error result in the execution results corresponds to which part of the error variables. However, existing fault localization methods based on statistics typically only need to know whether the software execution process is successful or not. Then collect two or more recordings during different execution processes, and calculate the fault possibility of code segment by statistical methods.

Fault localization based on program slicing is a representative of single execution record methods. Incremental debugging method based on coverage can fall into the fault localization method based on statistics.

CBFL (coverage based fault localization) method first inserts additional code, characterizing the behavior of software into the source code, and the program is called Cartridge. Then, execute the test cases and collect execution record. Finally, calculate the value of the defect tendency by counting the successful and failure rate in the process of a number of executions (commonly known as code coverage). Then isolate and sort code by the value based on debugging tendency, which narrows the scope of the code to be checked. Representative methods of CBFL are Tarantula [6], SBI [6], JACCARD [7], and OCHIAI [8].

Incremental fault localization method is called Delta Debugging; first by simplifying and separating the input method get a practicable test case, which has the closest running track with a failed test case. Then locate the fault in the program by comparing the two test cases' states at some point.

Program slicing based fault localization method, first proposed by Mark Weise, introduced the concept of the program slicing [9, 10] and the first static slicing algorithm and applied to the auxiliary debugger. A static slice represents a statement collection possibly affecting the output in the program [11]. Because debugging often needs to analyze the statements actually run in the program, dynamic slice is defined as a subset of statements set run in the program. It refers to the statements collection really impacting the output during the run. For dynamic slicing, the effectiveness of fault localization is judged by hit rate and searching scope.

In conclusion, the root of CBFL methods' defects is to ignore control dependencies and data dependencies between different statements in the program. So it will be a good choice to process the code by dependencies between programs and then locate faults by CBFL. While the root of program slicing fault localization methods' defects is a too large amount of defect codes, it is necessary to reduce the size of sliced codes appropriately. Research shows that, in sliced codes, some statements are not necessary. That is to say, there is no dependency between them and the expected output. Then we can consider reducing the sliced code amount by finding out these independent codes. The improvements of these two methods are required for analyzing the structure of the program and clearing the control dependencies and data dependencies between programs. So it is necessary to introduce the contents of analyzing the program structure in the following subsections.

## 3. Data Dependencies Analysis

### 3.1. Definition of Data Dependencies

For different types of code statements, we set different data dependencies.

**Figure 1:**
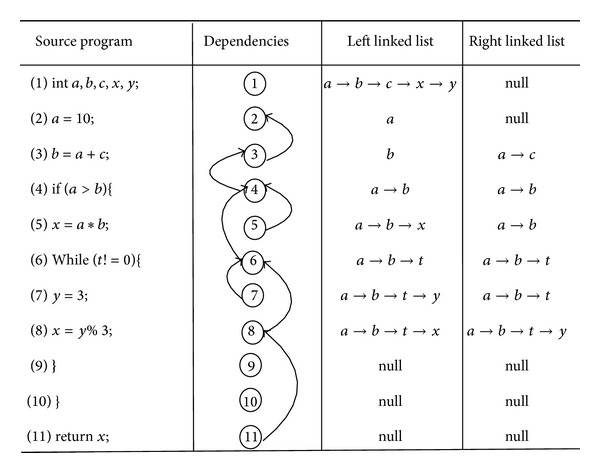
An example of data dependencies definition.

From Figure 1, we know that the left value and the right value are defined as two linked lists. For different types of statements, we define different data dependencies. For different code statements, we define different data dependencies, too. Specific code definitions are shown in Table 1.

### 3.2. Data-Dependent Extraction

We focused on reverse data dependence analysis on the same program execution paths and extract the data dependencies on this path. Based on a particular variable, we traversed the stored data dependence reversed.

#### 3.2.1. Data-Dependent Extraction Algorithm

Before reversing data dependencies analysis, it is essential to get different execution paths of the program based on analysis of control flow and then merge them.

Objects of reverse data dependencies extraction are execution paths extracted. Extraction process is along execution paths from back to front. Store the data dependencies before extracting reversely. The process of data dependency analysis and storage is actually doing text analysis of the program. Get types of code statements by lexical analysis and syntax analysis. Then gather left and right values of code segments to form linked lists, depending on the data dependencies on different code statements, in accordance with different ideas of extraction. At last, store the linked lists into data dependencies table. Specific extraction algorithm is as Algorithm 1.

The algorithm describes the execution algorithm of data dependencies extraction. The concrete implementation of the algorithm is given in the form of pseudocode. Different code types have different ideas of extraction and should be classified in detail.

#### 3.2.2. Data-Dependent Storage and Traversal Algorithms

**Figure 2:**
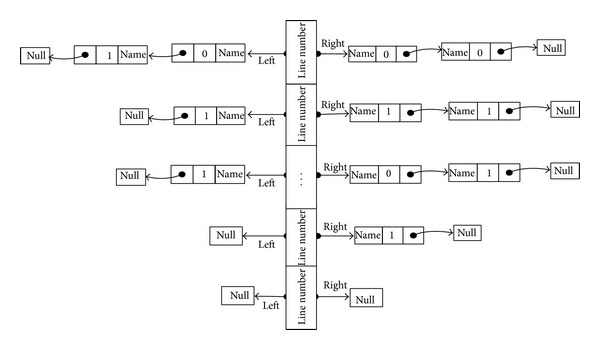
Data-dependent storage diagram.

In view of the definition of a data dependency and for subsequent traversal looking for convenience, we designed a data-dependent storage structure as Figure 2.

We define the basic node as a structure array, which is used to store each code line. This structure contains two fields, which are the left and right pointers, and link to each of the lines of code around value chains. It also contains a code line number, in order to search in the subsequent traversal store table easily. Each node consists of three fields, including the variable name of the node name, node type, and flag (value of 1 indicates a function and a value of 0 indicates normal variable), as well as a link to the next pointer field of the node next.

**Figure 3:**
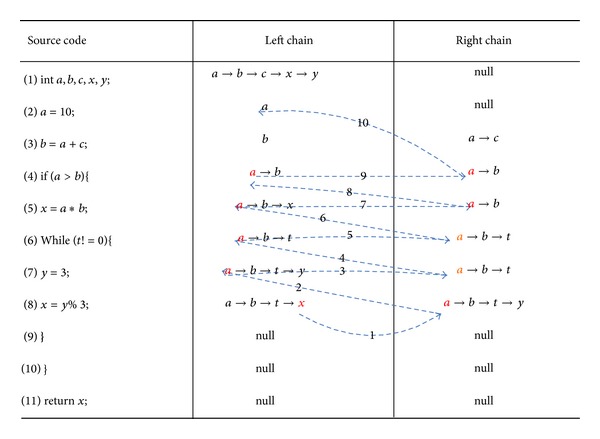
Data-dependent traverse diagram.

In the following section we will show how to traverse to find the specified variable. We use Figure 3 to illustrate basic search process.

As indicated in the figure, we specify the variables *x*; beginning to find from the last row of the stores table, find the row to *x* as an *l*-value (8 lines); line 8 is added into *S* collection, and collection *S* started with empty value. Then start to search from each variable of line 8 right chain in turn. Take the right chain of a variable *a* as an example, we specify *a* as a new variable, then we search the line ahead. The search process is exactly as *x*. We put the searched line number into *S* collection until all line numbers are found for the specified variable. Then, we find *b* to specify the line number variables. Until the reverse traversal to the row of right meets* null* values, it marks traverse to the end on the specified variable. This is a recursive lookup process. The figure only shows the first step to find variable *a*, without recursive lookup process. At the end of the first step of variable *a*, set *S* = {8,7, 6,5, 4,2}.

Until we find all variables in the right chain of line *x*, we traverse the collection s and eliminate duplicate rows to ensure uniqueness of the collection elements. The traversal algorithm is shown in Algorithm 2.

## 4. Coverage and Program Structure Slicing Fault Localization

### 4.1. Basic Idea Description

Considering the software security flaws and advantages of both CBFL and program date slicing fault localization and combining with the theoretical basis of the analysis of program structure, we propose a fault localization solution CPSS (coverage and program structure slicing) based on program execution path structure data dependency analysis.

**Figure 4:**
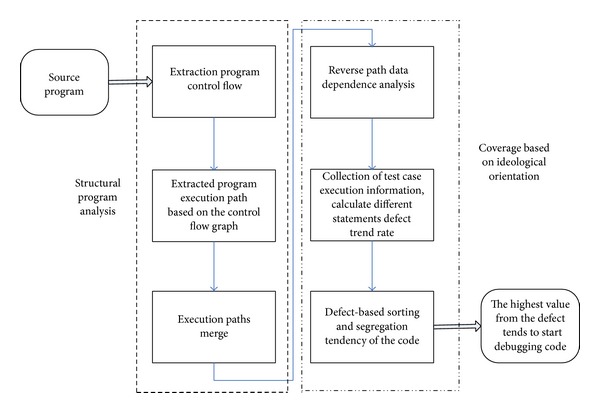
Basic idea description block diagram.

The basic idea description is as shown in Figure 4.

Extraction execution paths: it is the major work of the control flow analysis, according to the execution of test cases, combining with the relevant command GCC, extract program execution path.

Execution path mergence: every execution integration path is streamlined into a complete program dependence graph, for convenient follow-up coverage of statistical analysis.

Reverse data dependence analysis: through the application of text analysis, it is an important task of the reverse data dependence model and extracts the data on the execution path dependency.

Calculating defect rate: combined with the statistical error correction of quantitative calculation model, programming calculation of different defects tends to code statements.

Sorting code and isolation: the high and low order is from the high to low value according to the defects.

**Figure 5:**
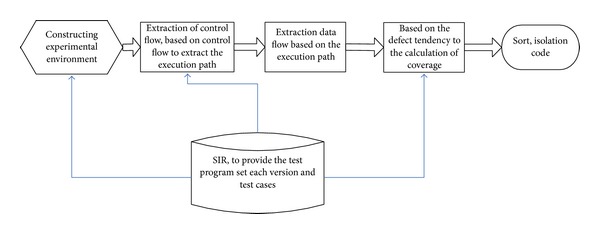
System structure diagram.

In order to more clearly show our error locating methods, this section gives a method of our overall execution system diagram as Figure 5.

Our research work mainly divides into five parts.

*(1) Constructing Experimental Environment Module.* Experiments involve SIR itemsets using the GCC command and the use of a shell script, so setup of appropriate experimental environment is the assurance that follow-up experiments can go smoothly. Set up the experimental environment; the main work is to set the environment variable to write a shell script, marking procedure, and hand all false and hand all errors and a series of work.

*(2) Extracting Program Control Flow and Extracting the Execution Path Module.* Based on the analysis of the control flow, we use GCC command to extract the program control flow diagram, based on the extraction of control flow file execution path of extraction by programming.

*(3) Extracting the Data Flow Module.* Through reverse data dependence model to extract the execution path of data dependency. We reduced the amount of code execution path by program slicing.

*(4) Quantitative Calculation Module.* Reference to quantitative calculation method, combining with the former several modules are program slicing, the locating method based on coverage, through the calculation of programming implementation code defects.

*(5) Ordering Module.* According to quantitative calculation module, result of a code defect level inclination value is as a basis for ordering code statement. Isolation of code statements tended to have a high priority in debugging.

### 4.2. Control Flow-Oriented Execution Paths Extraction and Mergence

It is vital to extract different execution paths of the program in reverse data dependencies process, and among them, extracting control flow, execution paths, and paths mergence are upmost basis of execution paths tracing.

Through comparing experiments on the program control flow graph above the execution results of the related test cases, we found that each test execution case corresponds to an execution path and failure test cases correspond to failure execution paths. And we also found out that the distribution of the failure of implementation errors in different control flow path is important for the improvement of positioning accuracy.

For example, suppose there are two basic block execution paths *e*_1_ and *e*_2_ covering *b*, *b*_1_ → *b*_2_ → *b*_4_ and *b*_1_ → *b*_3_ → *b*_4_, respectively. Set override basic block *b* process in the implementation of the following two conditions.

The first case, all cover *b*_1_ → *b*_3_ → *b*_4_, is successful implementation, all cover *b*_1_ → *b*_2_ → *b*_4_ is failed implementation, and the successful and failure implementation perform the same times as follows:

(1) failed (b1⟶b2⟶b4)=2Passed (b1⟶b2⟶b4)=0failed (b1⟶b3⟶b4)=0passed (b1⟶b3⟶b4)=2.

For the second case, cover *b*_1_ → *b*_3_ → *b*_4_ path of execution, implementation times of successes and failures are the same as follows:

(2) failed (b1⟶b2⟶b4)=1passed (b1⟶b2⟶b4)=1failed (b1⟶b3⟶b4)=1passed (b1⟶b3⟶b4)=1.

The basic code and the successful execution of the block *b* have the number of the same failures. For the first case, since *b*_1_ → *b*_3_ → *b*_4_ execution failure does not exist, the error status of basic block *b* does not pass the *b*_1_ → *b*_3_ → *b*_4_ delivery. If basic block *b* is flawed, *b*_1_ → *b*_3_ → *b*_4_ execution failure should exist. The same reasoning applies to the first case, the basic block *b* flawed great possibility.

The same control flow for different execution statistics is just double-counted, but different control flow paths of execution paths will fail to solve the program, which has a positive effect.

Therefore, By considering multiple execution paths in the actual testing process result, we merge different paths according to their corresponding control flow after all execution paths data are preprocessed.

## 5. Experimental Results and Analysis

### 5.1. Comparative Analysis of the Fault Localization Effect

In this section, we will take advantage of method CPSS proposed in our paper to locate the error in project flex v1 [12] to verify the effectiveness of the proposed method. We will compare the results obtained by using Tarantula, CT, and SBI.

To judge the CPSS's seeking effect, we need to contrast the result with the existing location methods. We chose three location methods, Tarantula, CT, and SBI. Specific experimental data are shown in Table 2.

Table 2 shows the respective proportional band of the detected code. Four methods are able to detect the percentage of faults. The abscissa is the ratio of code required to be detected to the total amount of codes. Ten percentage points represent that how much code is detected of each method. The vertical axis is the proportion of detected fault, which indicates what percentage of faults can be located in the abscissa specified levels. Under 20% of the code detection situation, CPSS can detect 85.71% of the faults, and other methods should be less than this figure. And only detecting 40% of the code CPSS can locate all faults; other methods cannot make it clearly. In the comparison of the remaining three methods, obviously, the SBI method is better than the other two methods.

Next, we will compare CPSS with SBI, the best method in the remaining, in different fault types, and analyze the pros and cons of these two methods in the location effects.

### 5.2. Comparative Analysis of SBI and CPSS Location Effect

According to the classification in Section 5.1, condition judgment fault, assignment fault, and function fault, this paper tries to compare CPSS with SBI and reveal the location effect of CPSS for further different fault styles.

#### 5.2.1. Comparison of the Condition Judgment Fault

Condition judgment fault refers to faults from if condition and condition judgment statement of while loop statement, and condition judgment is stricter or looser to make results not matching the expectation. Project flex v1 has 4 condition faults.

**Figure 6:**
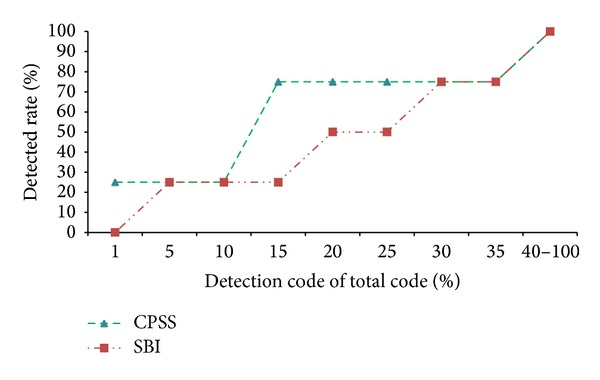
Comparison of location effect of condition judgment fault.

As can be seen from Figure 6, under the detection rate of 1% which can best embody the location effect, CPSS may locate 25% of condition faults; that is, the CPSS can accurately locate 25% of the condition faults, while SBI method cannot directly and accurately locate any condition fault. If the detection rate is 20%, CPSS may locate 75% of faults, but SBI method may locate only 50%. Within the range of 1%–30% of code detection, CPSS curve has been higher than the SBI curve, which means at this stage the effect of CPSS is better than SBI. Until the detection rate is greater than 30%, do they overlap with each other? In both after 40% of the code is detected, the detection rate reaches 100%; that is, detecting 40% of the code can fully locate all the condition faults, and the result can be accepted. Taken together, CPSS method is better than the SBI method when locating condition faults.

In many cases, the formation of condition fails because the values do not meet desired objectives. But CPSS method combines reverse data dependency model. During the reverse data dependence analysis process, we start from the end of the data stream that variables carry, reverse lookup step-by-step, analyze the change of variable values, and trace the source of faulted variables. This leads to the advantage of our CPSS method in condition fault localization.

#### 5.2.2. Comparison of the Assignment Fault

**Figure 7:**
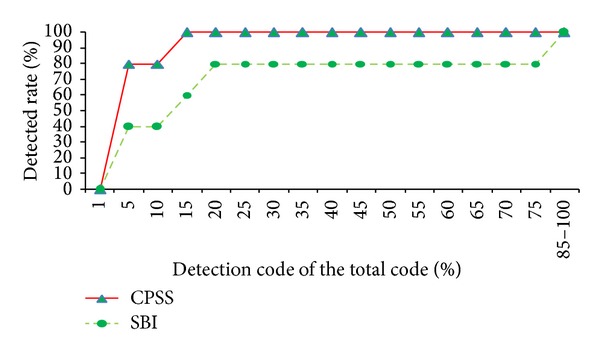
Location effect of the assignment fault.

What causes the assignment fault is the result inconsistent with the expected values because of miscalculation or wrong type of assignment. The fault localization effect of CPSS and SBI methods is shown in Figure 7.

As can be seen from Figure 7, with the allowed detection rate 1%, the ordinate values of CPSS and SBI methods curve are both 0%; that is to say, they are unable to directly and accurately locate the assignment faults. However, when the detection rate reaches 5%, the localization ratio of the CPSS method is quickly increased to 80%, but the SBI method is 40%. It can be roughly considered that the difference of location effect is half. With code detection rate of 15%, CPSS may locate 100% of faults, while SBI method only locates 60%. So it can better reflect the advantages of CPSS method in locating assignment faults. Figure 7 also shows that, in the range of 0%–80% of the code detecting, the CPSS curve has been higher than the SBI method. And only detecting 15% of the code CPSS can fully detect assignment faults, while SBI needs to detect 80% of the code, so CPSS method is obviously better than SBI method in assignment faults location.

Abstractly, the program code is only the carrier of the internal data stream, but the control structure is the framework of the program. Essentially, the reason of the assignment fault is that the correct flow of the program data stream goes wrong. Thus, the flow of data changes from the expected values. The location effect of CPSS method better than the SBI method is rooted in: the CPSS method does the control flow analysis and data flow analysis and extracts the data dependencies of the program, before collecting coverage rate and calculating. Discover the cause of faults deeply from the fault occurring, and pick up the code causing faults to search, so there is no doubt that it greatly narrows the scope of the code detection. It can locate the fault conveniently and quickly.

#### 5.2.3. Comparison of the Function Fault

Function faults are caused by the difference between the result created by wrong parameter passing and type mismatching in the function call process and the expected values.

**Figure 8:**
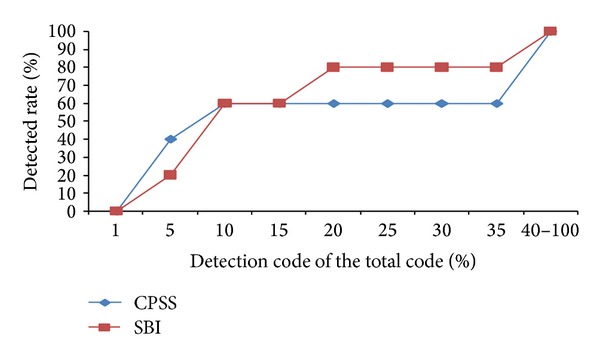
Location effect of the function fault.

From Figure 8, we can see the two methods in the process of locating function faults, having respective advantages in location effect. When the detected code is less than 10%, the CPSS method curve is higher than SBI; that is, the CPSS method is better than SBI. In the stage of 15%–40%, the SBI method is better than CPSS. Figure 8 also shows that, when the detection rate is 1%, faults detected account for 0% of the total number, indicating that neither CPSS nor SBI method is able to locate faults accurately and directly. With code detection rate of 40%, both curves reach the highest point of 100%, and then curves trend stably parallel to the *x*-axis; that is, almost all the function faults can be detected with 40% of the code detection rate. All in all, each of the two methods has its advantages in the function fault localization, but when the detection rate is very low, the location effect of CPSS is better than SBI's. A good fault localization method needs to have a low code detection rate but is able to detect a high percentage of faults. According to this evaluation standard, we can loosely think CPSS is little better than SBI.

## 6. Conclusion

Software fault localization and diagnosis are a critical step in the software debugging. Automated fault localization method can help developers to quickly find the location of the program fault and improve the efficiency of development. In this paper, based on Reverse Data Dependence Analysis Model to extract data dependencies between program statements by analyzing the advantages and disadvantages of the more generally applicable CBFL and program structure slicing method, for shortcomings, we proposed a new fault localization method CPSS while retaining the advantages.

## Figures and Tables

**Algorithm 1:**
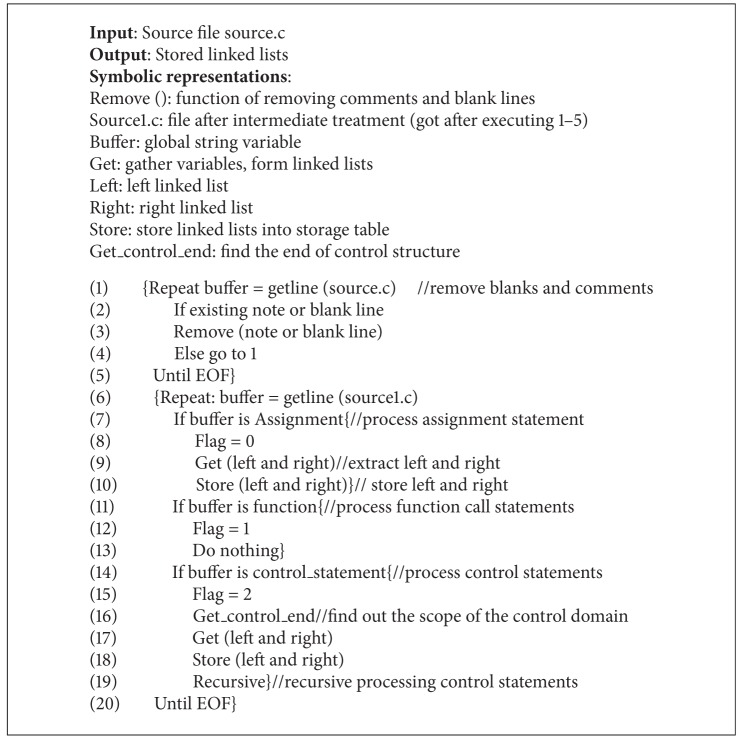
Data_dependent_extraction algorithm (source.c).

**Algorithm 2:**
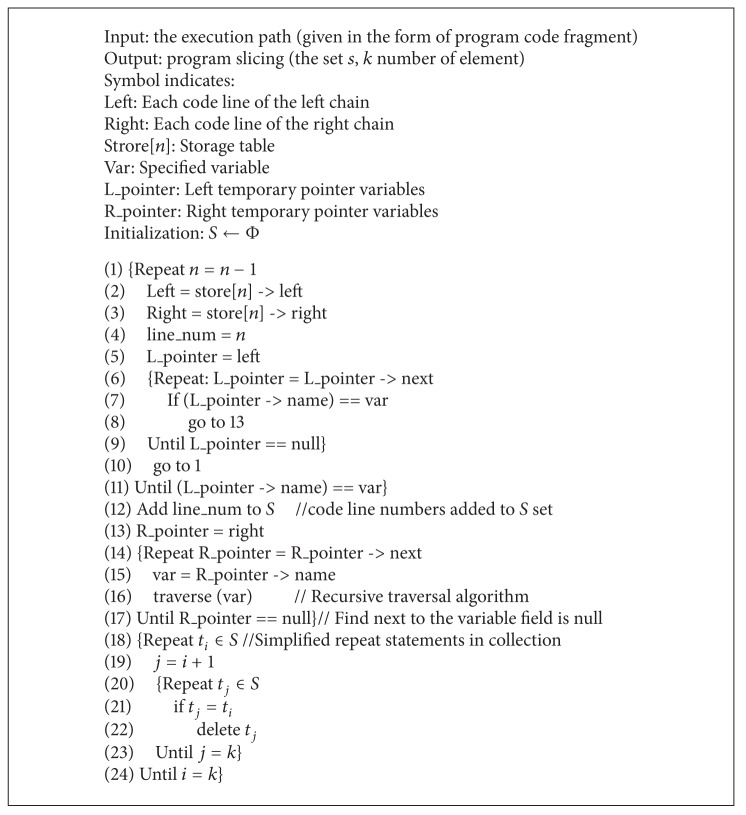
Traversal algorithm: traverse (var).

**Table 1:** Definitions of different statements data dependencies.

Statement type	Definition of left and right values	Examples of statements	Left and right linked lists	
Assignment statement				
Definition declaration	Left value is the linked list defining variables, and right value is null.	Int *a*, *b*;	*a* -> *b*	*a* -> *b*
Direct assignment	Left value is the linked list defining variables, and right value is null.	*a* = 10;	*a*	*a*
Indirect assignment	Left value is the assigned variable, and right value is the assigned value.	*a* = *b* + *c*;	*a*	*b* -> *c*
Control statement				
While	Left value and right value are the same in these statements, which are variables involved in(). Left and right values of these statements are included in the left and right linked lists of all statements in the control domains.	While (*a* > *b*)	*a* -> *b*	*a* -> *b*
If else		If (*a* > 10)	*a*	*a*
For		For (*i* = 1; *i* < *b*; *i*++)	*i* -> *b*	*i* -> *b*
Console statement	Left value and right value are both null.	return *x*;	Null	Null
Function call				
User-defined	Related to parameter types.	Abs(∗*a*, *b*)	*a*	Abs()
Library function	Left and right values are both null.	Print f()	Null	Null

**Table 2:** Within the scope of each code to detect the error ratio.

Testing code rate%	CPSS	Tarantula	CT	SBI
1	7.14	6.93	5.26	7.14
10	50.00	47.36	26.36	50.00
20	85.71	63.16	36.84	71.43
30	92.86	71.31	51.17	78.57
40	100.00	79.51	52.72	92.86
50	100.00	86.89	59.70	92.86
60	100.00	87.71	62.80	92.86
70	100.00	88.53	70.55	92.86
80	100.00	92.63	75.20	100.00
90	100.00	100.00	82.18	100.00
100	100.00	100.00	100.00	100.00
